# A scoping review of evidence on routine cervical cancer screening in South Asia: investigating factors affecting adoption and implementation

**DOI:** 10.1007/s10552-024-01923-y

**Published:** 2024-10-07

**Authors:** Priyobrat Rajkhowa, Mebin Mathew, Razeena Fadra, Soumyajit Saha, K. Rakshitha, Prakash Narayanan, Helmut Brand

**Affiliations:** 1 Department of Health Policy, Prasanna School of Public Health (PSPH), Manipal Academy of Higher Education (MAHE), Manipal, 576104 Karnataka India; 2 Department of International Health, Care and Public Health Research Institute – CAPHRI, Faculty of Health Medicine and Life Sciences, Maastricht University, Maastricht, The Netherlands; 3 Department of Global Health Governance, Prasanna School of Public Health, Manipal Academy of Higher Education (MAHE), Manipal, Karnataka 576104 India

**Keywords:** Cervical cancer screening, CFIR, Adoption and implementation, South Asia

## Abstract

**Need:**

Cervical cancer is a major global public health issue, particularly affecting low and middle-income countries, distinctly in the South Asian region. This geographical region lacks a well-organized routine cervical screening program. Consequently, this scoping review aimed to investigate the evidence on factors influencing the adoption and implementation of routine cervical cancer screening in South Asia.

**Methods:**

Adopting the “Arksey and O’Malley and Levac et al.” methodology, databases such as PubMed, CINAHL, Web of Science, and Scopus were scrutinized in the pursuit of relevant studies. Subsequently, the collected data were synthesized by adopting the Consolidated Framework for Implementation Research (CFIR) model.

**Results:**

A total of 837 records were initially identified and screened for eligibility, including 55 studies. The successful adoption and implementation of cervical cancer screening in South Asia encounter numerous obstacles within the health system, including the absence of a comprehensive program protocol for screening, inadequate health infrastructure, and the presence of multiple sociocultural factors, such as social stigma, low levels of education, and concerns related to modesty.

**Conclusion:**

To optimize adoption and implementation, it is imperative to construct a customized policy framework that incorporates a risk communication strategy tailored to the specific contexts of these nations. Drawing insights from the experiences of South Asian countries in executing cervical cancer screening programs can inform the formulation of policies for similar healthcare initiatives aimed at facilitating the expansion of HPV vaccination efforts.

**Supplementary Information:**

The online version contains supplementary material available at 10.1007/s10552-024-01923-y.

## Introduction

Cervical cancer is a major public health issue worldwide, resulting in premature mortality and disability. The GLOBCON Report 2020 reveals an increasing trend in the incidence of cervical cancer, with 604,127 cases and 341,831 deaths reported globally [1]. The Asia-Oceania region, specifically Southeast and South-central Asia, has the highest incidence and mortality rates for cervical cancer [2, 3]. The majority of cervical cancer cases occur in low and middle-income countries (LMIC) that lack organized screening and Human papillomavirus (HPV) vaccination [4]. In South Asia, India bears a disproportionately large share of the burden, accounting for 15.2% of cervical cancer fatalities worldwide [5]. To tackle this pressing health challenge and eliminate cervical cancer as a significant burden from LMIC, the World Health Organization (WHO) has proposed a “triple-intervention coverage” strategy that involves targets for scale-up of HPV vaccination to 90%, twice-lifetime cervical screening to 70%, and treatment of pre-invasive lesions and invasive cancer to 90% [6, 7]. This approach is particularly relevant for South Asian countries, where most nations are classified as LMIC [8].

Multiple preventive measures, including HPV vaccination and routine cervical screening, can be adopted to halt the rising cervical cancer statistics [9]. However, South Asian countries have insufficient HPV vaccination coverage, and even with adequate coverage, vaccines cannot fully protect against the disease in LMIC [10]. Early prevention through screening is essential as cervical dysplasia can progress to carcinoma [11, 12]. Screening interventions provide an opportunity for early detection and prevention, potentially reducing mortality from cervical cancer. Consequently, the comprehensive adoption and implementation of routine cervical cancer screening is essential to eliminate cervical cancer as a public health issue in LMIC, particularly in South Asia.

The CFIR model is a theoretical framework that aims to comprehensively identify and understand factors that may impact the implementation of an intervention within [13]. The model comprises five major domains. The first domain, Intervention Characteristics, concentrates on the properties of the intervention itself. The second domain, the Outer Setting, includes factors in the broader external environment that may influence the implementation process. The third domain, Inner Setting, comprises elements within the organization or setting where the intervention will be implemented. The fourth domain, Individuals, considers the characteristics of the individuals involved in the implementation process. Finally, the fifth domain, the implementation process, focuses on the specific strategies and methods used to implement the intervention.

The CFIR model has been utilized in different health contexts, such as mHealth adoption by cancer patients and school sexual assault prevention programs [14, 15]. However, this study is the first to adopt the CFIR model in the context of routine cervical cancer screening specifically in Southeast Asia. This scoping review examines the evidence on factors influencing the adoption and implementation of routine cervical cancer screening in South Asia. This study identified the gaps in the health system that need attention to improve the implementation or strengthening of cervical cancer screening services. Summarizing existing evidence provides an overview of cervical cancer screening practices and supports further research for informed decision-making and interventions. Therefore, it is crucial to identify the barriers preventing women from utilizing routine cervical cancer screening services to implement or strengthen a successful program.

## Methods and analysis

The scoping review adopted the “Arksey and O’Malley and Levac et al.” methodology [16]. To ensure adequate coverage of necessary elements in reporting, the “Preferred Reporting Items for Scoping Reviews (PRISMA-ScR)” was adopted [17], and its recommended items are included in Online Annexure 1. The subsequent sections elaborate on the specific stages employed during this scoping review.

**Stage 1: Identifying the review question:** The team brainstormed and refined to come up with the review question. What factors influence routine cervical cancer screening adoption and implementation among South Asian women?

PCC (Population, Concept, Context) format, according to the JBI manual for evidence synthesis 2020 (Refer to Table 1), has been adopted for developing the research question [18].

**Table 1:** PCC framework for developing the research question

Population (P)	Women (35 to 45 years of age) [7]
Concept (C)	Factors associated with cervical cancer screening
Context (C)	South Asia (Afghanistan, Bangladesh, Bhutan, India, Maldives, Nepal, Pakistan, Sri Lanka) [19]

**Stage 2: Study identification:** The research question was divided into concepts, and relevant keywords were identified for each concept. The searched databases included PubMed, CINAHL Complete, Web of Science, and Scopus, with a limited scope to only English-language articles. An overview of the electronic search in the databases is provided in Online Annexure 2. In addition to the database search, reference lists of included papers were screened to identify any potentially qualifying articles. Furthermore, gray literature was identified and included through thorough searches of Google Scholar and by reviewing the reference lists of the included studies.

**Stage 3: Study selection:** The titles and abstracts of the retrieved papers were reviewed by four reviewers (PR, MM, RF, and SS) using the Rayyan platform [20]. If there was uncertainty about a study’s eligibility, the fifth reviewer (RK) provided their opinion. After the initial screening, the full texts of the eligible studies were reviewed by consensus among the five reviewers (PR, MM, RF, SS, and RK).

Based on the specified inclusion criteria, all studies that meet the requirements outlined in Table 2 were chosen for this review, regardless of their quality or rigour. This review includes various original research methods, such as quantitative, qualitative, and mixed-methods studies. Additionally, to ensure thorough coverage, the reference lists of the included papers were carefully examined to include all relevant literature.

**Table 2:** Inclusion and exclusion criteria

Inclusion criteria	Exclusion criteria
Studies focused on factors related to cervical cancer screening implementation/delivery and uptake/acceptance	Studies focused on cervical cancer primordial preventive measures, vaccination, treatment, and rehabilitation
Studies from South Asia	Studies focused on any gynaecological screening other than cervical cancer
Studies published in the English language	Studies forced only on the efficacy of a screening test

**Stage 4: Charting the data:** Data charting was performed by adopting a pre-defined format (Online Annexure 3). Each article included in the review was independently charted by five reviewers, PR, MM, RF, SS, and RK, using Microsoft Excel spreadsheets. In cases of disagreement, a sixth reviewer was consulted (PN). The charting process covered various aspects such as participants, context, concepts, study methods, facilitators, barriers, and critical findings relevant to the review questions. To meet these objectives, data extraction from the selected studies was completed. The data extraction form, detailed in Online Annexure 3, was developed and used to capture this information. If additional or missing information was needed, the authors of the papers were contacted.

**Fig. 1:**
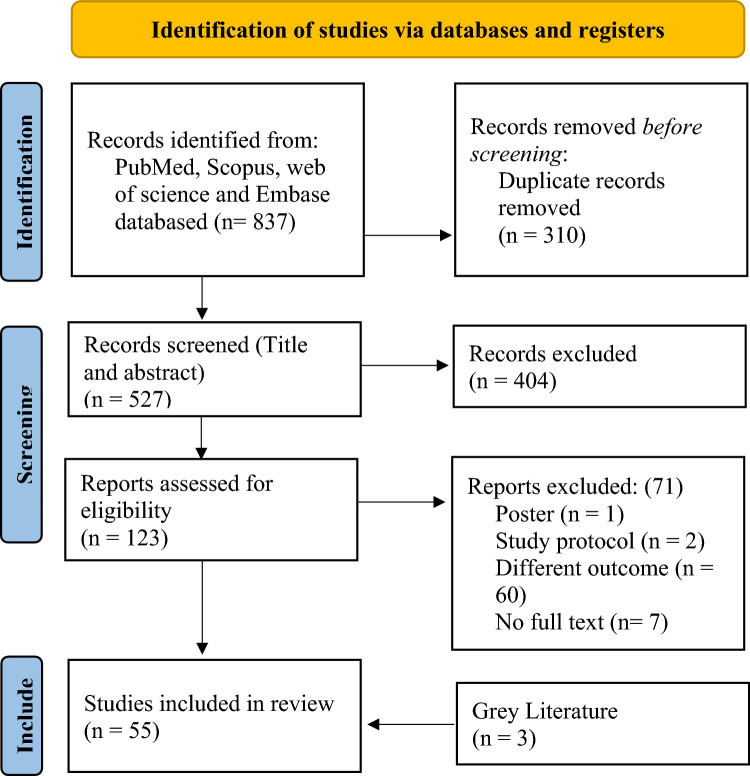
PRISMA Flow diagram of the factors influencing scoping review

**Stage 5: Collating, summarizing, and reporting the results:** The investigation findings were summarized using narrative synthesis and presented in a table. The data from the table were used to analyze the literature on cervical cancer initiatives in South Asia. The review includes a comprehensive report on the search results and study selection process, presented in a flowchart using the PRISMA 2020 flow diagram Fig. 1 [21].

## Result

### Characteristics of the included studies

This study conducted a thorough search, resulting in 527 unique entries after removing duplicates (Fig. 1). These entries underwent strict screening based on eligibility criteria, leading to the inclusion of 55 studies, including three gray literature. The review encompassed Bangladesh, India, Nepal, Pakistan, and Sri Lanka. A detailed summary of each investigation is provided in Table 3 is included in Online Annexure 4.

The success of a health program relies on how it’s adopted and implemented. Understanding these factors is vital for program success. Various aspects that impact the adoption and implementation of such programs in the region have been identified. These factors include inadequate targeting of the right age group, women’s hesitance towards cervical screening, and sociocultural and ethical barriers to sexual health, additionally, these factors have been described according to the countries, as highlighted in Table 4, Online Annexure 4.

**Fig. 2:**
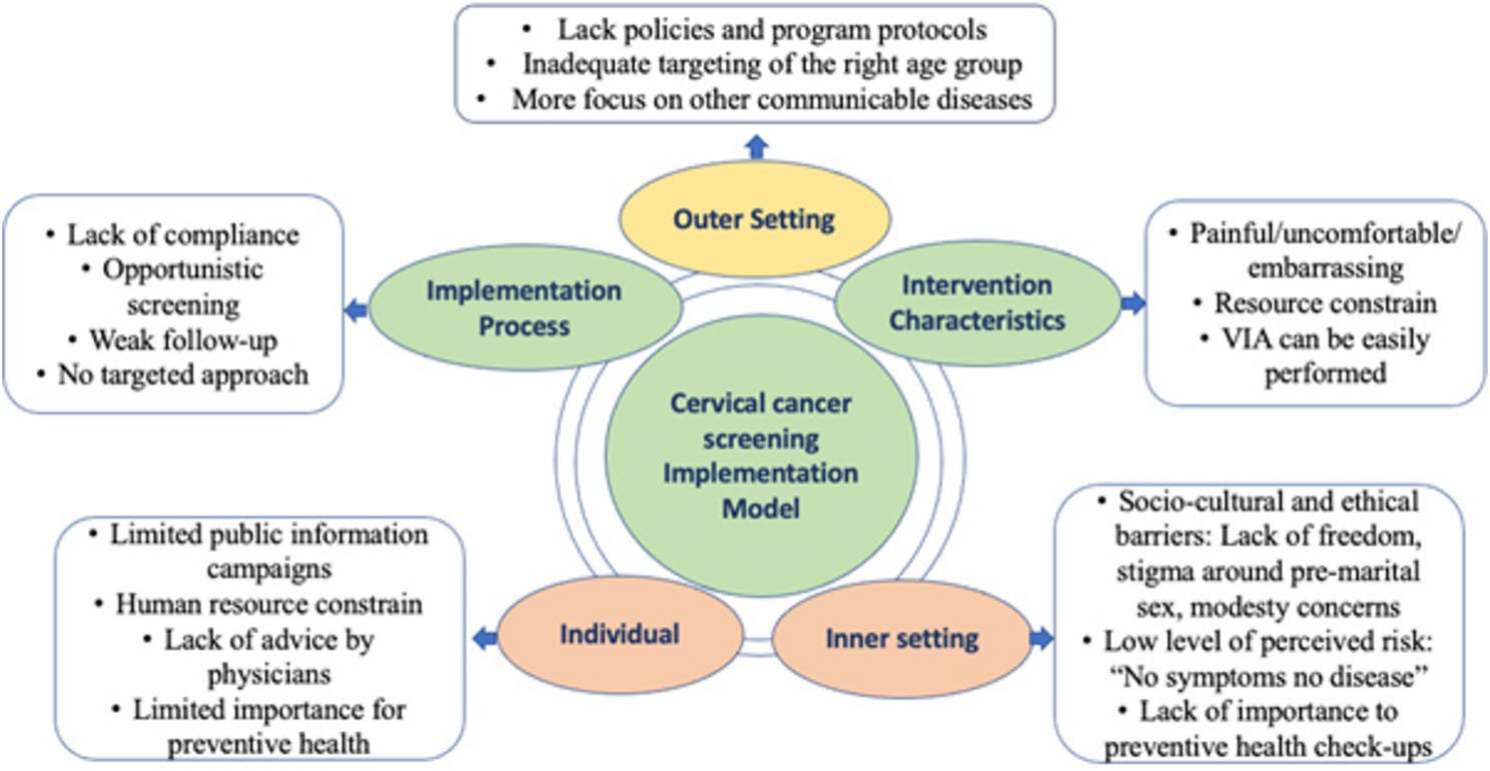
Adopted CFIR domains, constructs, and subconstructs of the cervical cancer screening implementation model, CFIR

The implementation and adoption of routine cervical cancer screening across South Asia are shaped by a range of factors Fig. 2. In Bangladesh, the availability of cervical screening services and the involvement of community healthcare workers have enhanced participation, though rural areas face challenges due to limited awareness, inadequate infrastructure, and cultural barriers. India shows progress through government initiatives, trained personnel, and increased awareness among women, but social stigma, financial constraints, and insufficient physician recommendations remain significant obstacles. In Nepal, the presence of low-cost screening methods and national guidelines facilitates implementation, although misconceptions and a lack of familial support hinder broader adoption. In Pakistan, while healthcare students demonstrate some awareness of screening, overall knowledge and attitudes toward cervical cancer prevention are inadequate, particularly in socioeconomically disadvantaged areas. Sri Lanka has benefited from awareness campaigns and media outreach, yet stigma, discomfort associated with screening, and limited participation among unmarried women persist as challenges. The following sections provide in-depth analysis of these factors by country.

### Bangladesh

Based on the existing body of literature, several favorable factors have been identified as influencing cervical cancer screening in Bangladesh. One such factor is the presence of opportunistic screening methods [22, 23]. Moreover, the screening uptake is positively influenced by higher levels of female education, urban residency, employment outside the home, and improved knowledge regarding cervical cancer and screening [22]. Furthermore, women in Bangladesh are aware of the availability of screening tests and exhibit a keen interest in utilizing screening services [24, 25]. Despite limited resources, VIA (Visual Inspection with Acetic Acid) can be conducted by trained medical professionals, including doctors, nurses, and paramedical workers in Bangladesh [25].

Further, it has been reported that the availability of advertisements, posters, and dissemination of screening programs by community health workers can improve the inflow of participants for screening [23]. The active participation of community health workers in disseminating awareness of the screening program, as well as partnerships with NGOs and religious and political organizations, have also been shown to improve the program’s efficacy [23]. Competency-based teaching curricula and training of a critical number of service providers are also essential for the success of the screening program [23].

In addition to the favorable factors, numerous challenges impede the widespread adoption and implementation of routine cervical screening in Bangladesh. Women residing in rural areas and possessing primary or no education are the most uninformed about cervical cancer and screening procedures [22]. This group of women is unaware of the need for screening due to a lack of symptoms or knowledge [22]. Additionally, cultural or religious beliefs further exacerbate the issue [22]. Another limiting factor for adoption is the low coverage of services among the target population [23]. Furthermore, inadequate adherence to these services poses a significant obstacle to effective adoption and implementation [23]. Moreover, healthcare providers have emphasized the insufficiency of health infrastructure in facilitating the service and have advocated for enhancements in infrastructure to accommodate the growing demands [25]. Additionally, the transfer of trained personnel has been identified as another concern by service providers, resulting in a scarcity of VIA-trained staff at multiple healthcare centres [25].

### India

Multiple factors contribute to the success of cervical cancer screening in India. These factors include the availability of resources, technology, and trained professionals, which increase awareness among low socioeconomic women and encourage screening adoption [26–28]. Women, mainly working individuals and healthcare professionals, have good knowledge about cervical cancer, screening, and vaccination [29–32]. Also, healthcare providers are willing to undergo screening training [31], and the attitudes of husbands and family members positively influence screening uptake [33].

Despite the social stigma, unmarried women also participate in screening [34]. Moreover, it has been found that using a simple and low-cost test, such as VIA, is acceptable to women [34]. Cervical cancer self-sampling is comfortable and can impact adoption [35]. Single-visit colposcopy-and-treat strategies have high compliance rates for immediate treatment [35].

The availability of mobile screening units can improve access and adequate resources available [36]. In contrast, VIA is safe, acceptable, and cost-effective, even in remote areas [37–39]. Additionally, screening can be conducted by paramedical staff [40], with women acknowledging these tests and not considering them as harmful [41], and women also express willingness to undergo screening [42–44].

Tests like Truenat HPVHR are suitable for community-level low-resource screening [45]. Government policies, mandatory programs, incentives, and awareness campaigns also contribute to increased screening and have been reported as enablers for increasing screening [46].

In conclusion, multiple favorable factors impact cervical cancer screening in India, including the availability of sources, technology, and professionals, the willingness of healthcare providers to undergo training, the attitude of husbands and family members, and the acceptance of simple and low-cost tests by women. Mobile screening units, community-level screening tests, and government policies promoting mandatory screening programs and creating awareness can also increase screening uptake.

In addition to the favorable factors, numerous challenges impede the widespread adoption and implementation of routine cervical screening in India. Multiple factors hinder the implementation and adoption of cervical cancer screening in India. These include a lack of awareness among low socioeconomic women [26, 29, 40, 42, 43, 47–53]. Potential social [34], cultural factors and taboo [29, 34, 42, 54, 55], limited availability of infrastructure and trained human resources [56], lack of advice by physicians [57], low perceived risk of getting the disease [52], absence of disease symptoms or family members/friends getting screened [28, 29, 32, 41, 49, 52, 58–60], lack of understanding preventive health check-ups [29, 41, 49, 52, 58], limited access to healthcare, psychosocial and cultural contexts [29, 41, 42, 61], social stigma [41, 49], lack of time [41, 49, 52], lack of public information campaign [36], high cost of HPV DNA and pap test [41, 62], household responsibilities and family-related challenges [42, 63], inadequate training for healthcare workers [61], difficulties in accessing screening due to costs and long travel distances [51], lack of specific orders and program protocols at the district level [51], Pain associated with the test, fear, modesty concerns, and financial constraints [52]. Fear of becoming isolated from family, lack of freedom to make one’s own decision, longer waiting hours for screening in the hospital, financial constraints, lack of financial incentives [52], symptom-based screening practices [40], decentralized healthcare system and limited resources [64], sociocultural beliefs, and economic factors [65], and limited access to private and public clinics [63].

### Nepal

Routine cervical cancer screening in Nepal is influenced by various factors, including the establishment of the National Cervical Cancer Screening and Prevention Guideline in 2010 [66]. Concerning the self-sampling kit, women were willing to accept it if the cost was reasonably affordable [67]. Additionally, individuals with a positive family history of cancer were more likely to participate in cervical cancer screening [68].

Multiple barriers impede routine cervical cancer screening in Nepal, including long waiting times, discomfort with male healthcare providers, fear of test results, and the perception that screening is unnecessary for healthy individuals [69]. The limited availability of screening services and a shortage of skilled personnel and equipment contribute to the low screening rate [70]. Lack of knowledge, myths, poverty, fear, limited accessibility, privacy concerns, and lack of support from family members also hinder participation [66]. Many women prioritize household needs over healthcare expenses, and urban Nepali women face challenges due to a limited understanding of cervical cancer and its link to HPV [67]. Most women in Nepal have never been screened for cervical cancer [68].

### Pakistan

In Pakistan, various factors affect the adoption and acceptance of routine cervical cancer screening. These include challenges like limited knowledge and negative attitudes among healthcare staff, socioeconomic disparities in certain regions, and low education levels [71]. Moreover, even healthcare students have an insufficient understanding of cervical cancer’s prevalence, although most are aware of the availability of screening tests, which may impact successful screening programs [72].

### Sri Lanka

Multiple factors influence the adoption and implementation of routine cervical cancer screening in Sri Lanka. One such factor is the significant role played by the media, as observed among individuals who were already aware of Pap smears [73]. Incorporating HPV/DNA screening as the primary method for cervical cancer screening can alleviate the workload of cytoscreeners and Consultant Histopathologists [74]. Education and awareness regarding these malignancies are sporadically acquired by the general public through various sources such as newspapers, magazines, the internet, and televised healthcare programs [75]. Implementing the new HPV/DNA test was highly appreciated by most women when accompanied by well-organized community awareness campaigns and effective field staff performance. Furthermore, satisfaction was expressed with the clinic facilities and the performance of clinic staff in conducting HPV/DNA screening tests [76].

Routine cervical cancer screening faces multiple obstacles, including inadequate coverage of Pap tests in Sri Lanka, even among female healthcare workers [73]. Factors like cultural influences and embarrassment hinder the response rate. Although women are aware of the Pap smear test, many lack an understanding of its purpose, indicating a gap between awareness and comprehension. Limited dissemination of health education and awareness campaigns contributes to the low awareness of Pap smear testing. The primary reasons for low uptake include sociocultural factors, inadequate understanding, and the absence of a call and recall system. In Sri Lankan culture, discussing or offering Pap smear testing to unmarried women may be avoided due to the stigma surrounding pre-marital sex, leading to lower participation rates [73]. Furthermore, no comprehensive community-wide educational program currently targets sexually active women of reproductive age [75]. The fear of discomfort during HPV/DNA screening also emerges as a significant barrier [76].

## Discussion

Cervical cancer screening plays a crucial role in preventing cervical cancer-related consequences. However, adopting and implementing routine cervical cancer screening programs in South Asia has encountered numerous challenges regarding acceptance and delivery. Drawing on the results of 55 studies, this study presents an overview of factors that have influenced the adoption and implementation of cervical cancer screening programs. These factors are classified and discussed according to the five primary domains of the CFIR model, as highlighted in Fig. 2.

A comprehensive evaluation of the external factors impacting the adoption and implementation process is essential to ensure effective adoption and implementation. This evaluation encompasses aspects such as the lack of a policy protocol, which plays a significant role in executing cervical cancer screening programs. Challenges in effective screening service delivery have been identified due to the absence of a specific screening protocol and inadequacies in healthcare staffing. To combat these obstacles, the WHO has endorsed a global strategy known as the “triple-intervention coverage” strategy, with the ambitious goal of eliminating cervical cancer by 2030 [7]. South Asian countries can adopt and adapt these strategies to develop a comprehensive screening protocol for their implementation. In India, the government is on the verge of deployment of the HPV vaccine targeting girls aged (21). The recent development of the HPV vaccine in India and its gradual adoption and implementation presents an opportunity to incorporate screening strategies and HPV vaccination [77]. Strengthening the healthcare system through Universal Health Coverage (UHC), implemented in 2015, can further support these efforts, potentially enhancing cervical cancer screening programs in South Asia [78]. Ultimately, these measures contribute to achieving the Sustainable Development Goals (SDGs). To address and mitigate these challenges, a balanced approach is crucial. Countries should utilize available information to develop strategies for effectively utilizing healthcare resources, particularly in South Asian countries where the lack of healthcare staff, inadequate infrastructure, and imbalanced distribution of health workers hinder healthcare services [79].

The successful adoption and implementation of cervical cancer screening necessitates meticulously evaluating intervention characteristics and their impact on adoption and implementation. This includes factors like accessible and affordable tests, community-based and self-sampling screening approaches, painful procedures, women find the tests embracing and decision-making processes for adopting screening techniques. Among these methods, the VIA/ Visual inspection with Lugol’s Iodine (VILI) test is a simple and sensitive technique [80]. Additionally, it offers the advantage of being easy to perform by any trained healthcare professionals and can be performed on-site screening and treatment for minor abnormalities [81]. This provides a promising opportunity to broaden screening services [82]. However, VIA has poor specificity, so it should not be solely relied upon as a see-and-treat method [76, 79, 80, 83, 84]. Therefore, WHO recommends HPV DNA detection as the primary screening test due to its effectiveness [85, 86]. These advancements in self-sampling methods offer a distinct opportunity to enhance the adoption of screening measures [87]. It can supplement reaching the global target of 70% screening coverage by 2030. Women may also feel more comfortable taking their samples than seeing a health worker for cervical cancer screening. In many healthcare settings, women have accepted self-sampling methods [88, 89].

Additionally, women have highlighted that the screening tests are embracing and can cause discomfort, leading to hesitancy towards such tests. Thus, self-sampling methods can potentially supplement screening services in South Asia. Women naturally may feel embarrassed and uncomfortable with any gynaecological examination [90]. Therefore, in places where HPV tests are available, healthcare decision-makers should consider the inclusion of HPV self-sampling as a complementary option within their existing approaches to address gaps in current coverage [85]. However, it is essential to note that this paper does not endorse any specific intervention method for addressing these factors.

In the context of the Inner setting, multiple sociocultural factors, such as stigma, taboos, family dynamics, education, marital status, and religious beliefs, significantly impact the screening process. Studies have shown that stigma leads to hesitancy in accepting screening due to misconceptions linking it to sexual promiscuity [91–93]. Instances have been documented where stigma-driven influences affect healthcare delivery [94]. Additionally, it weakens social cohesiveness, which could result in social isolation, and people might be reluctant to reveal or minimize the frequency or severity of their symptoms, which makes disease prevention more difficult. Stigma has negative consequences on mental health and has led to non-compliance with medical advice, as evidenced in cases of HIV and COVID-19 [95, 96].

Furthermore, stigma has been observed to reduce testing rates for other stigmatized diseases [95]. These beliefs have the potential to impede progress towards the goal of eliminating cervical cancer, necessitating the implementation of a risk communication plan to strengthen community healthcare delivery. Due to the stigmatization surrounding HPV, the virus associated with cervical cancer, and the common assumption that it primarily affects sexually active women, people have exhibited non-compliance towards screening. These access limitations have also been observed in other stigmatized conditions [97]. Given that women’s health is often marginalized [98], achieving effective implementation would require a deep understanding of its technical components, as it is a subject that must be researched and approached with informed decision-making in South Asia. A targeted empowerment program is essential to enhance women’s autonomy in household decision-making and improve participation in cervical cancer screening, considering the lack of spousal and familial support in decision-making situations [99]. A social science investigation is warranted to address disparities in women’s autonomy based on education, wealth quintile, and development region. Implementing a comprehensive women empowerment strategy can effectively enhance their decision-making autonomy.

The concerns related to compliance with routine cervical cancer screening are also closely linked to the potential loss of income experienced due to indirect costs such as travel to distant healthcare facilities. This kind of pattern has been observed in various contexts [100]. Notably, interventions like the Janani Suraksha Yojana, which provides financial incentives for utilizing services, have shown effectiveness [101]. Implementing a similar policy approach could involve offering incentives to encourage participation in cervical cancer screening programs. However, ensuring that the benefits are distributed equitably and should not disproportionately favor wealthier groups is essential, as previous research has documented such disparities [102].

Factors such as knowledge, attitudes, beliefs, and women’s motivation are essential in influencing cervical cancer screening adoption. However, barriers such as lack of awareness and insufficient knowledge exist, leading to low compliance with screening practices. Similarly, the general population’s limited understanding and awareness regarding the importance of health services have consistently been associated with low uptake rates. Moreover, the lack of awareness among the general population can contribute to reduced utilization of health services [103]. Therefore, a well-structured health promotion strategy tailored for South Asian women, aligned with the Sustainable Development Goals, is crucial [104]. The opportunity provided by the Ayushman Bharat Programme launched in 2018 should be leveraged in India and other countries with similar health programs for rapid expansion and adequate coverage of cancer prevention and treatment interventions in India.

To ensure the successful adoption and implementation of cervical cancer screening, it is imperative to conduct a comprehensive evaluation of the individual factors that may impact the implementation process. The Active involvement of healthcare professionals is crucial for promoting cervical cancer screening, but barriers like insufficient training exist. Studies show that increased training improves knowledge and skills, leading to successful vaccination goals [105]. To address this, programs such as implementing WHO-recommended training programs for healthcare workers are urgently needed [106]. Motivation is another factor that affects the adoption of routine cervical cancer screening, as women often fail to recognize its significance unless they have symptoms. Consistent encouragement and active involvement of healthcare professionals are necessary to engage women in screenings [107]. However, it has been previously emphasized that healthcare workers require adequate training to conduct these screening services. Nevertheless, it is crucial to recognize that heightened awareness among healthcare providers can contribute to accomplishing healthcare objectives [108]. Therefore, there is an urgent need to implement screening communication strategies that enhance understanding and encourage the adoption and delivery of cervical cancer screening.

In conclusion, the domain of the implementation process has identified planning, training, weak follow-up, availability of opportunistic screening only, and monitoring as crucial elements for achieving successful adoption and implementation. Scientific evidence supports providing training and continuing education courses to health professionals for effective healthcare delivery [109]. The predominant approach for adopting and implementing the HPV vaccine is school-based vaccination, which has demonstrated success [110]. However, when combining HPV vaccination with screening, there is a significant difference in the target age groups, which may pose challenges in integrating these services. HPV vaccination is typically administered in a school health setting, while screening for adults can only be done in healthcare facilities. This raises questions about the feasibility of adopting the WHO-suggested triple-intervention strategy for countries facing these challenges. In this field, females often show non-compliance with screening, and cancer registries in these nations are limited [111], indicating a need for their improvement. Consequently, it is imperative to foster the enhancement of cancer registries while concurrently devising tailored methodologies to mitigate attrition rates. The lack of a dedicated policy for cervical cancer elimination hinders structured screening initiatives, leading to reliance on opportunistic screening.

The investigation of factors influencing cervical cancer screening adoption and implementation is crucial. Understanding and addressing barriers to adoption and implementation is necessary for practical solutions. Findings can guide policy recommendations to overcome obstacles. It is essential to recognize that implementing or strengthening cervical cancer screening in South Asian countries requires tailored strategies due to unique challenges in each country. However, leveraging favorable factors can promote widespread adoption in South Asia. Empowered communities and healthcare personnel can collaborate to modify stigmatizing healthcare policies [112]. Developing a risk communication plan is essential to combat disease-related stigma. Risk communication strategies have effectively addressed stigma in diseases like HIV/AIDS [113]. In low-resource nations, the recent advent of a domestically produced HPV vaccine in India is expected to decrease costs associated with vaccine doses and enhance implementation efforts of cervical cancer screening combined with vaccination [114]. Lessons learned from these experiences can be utilized to expand the scale of the country’s cervical cancer screening programs, thereby contributing to the target of eliminating cervical cancer and attaining sustainable development goals.

In conclusion, adopting and implementing routine cervical cancer screening in South Asia, systemic and sociocultural hurdles must be removed throughout South Asia. A feasible route to elimination by 2030 is to incorporate the HPV vaccine and implement the WHO’s “triple-intervention coverage” strategy. Reducing societal stigmas, promoting women’s independence, and giving medical staff thorough training are essential for success. The use of tailored risk communication and self-sampling techniques will encourage the broader uptake of screening. Together, these initiatives can significantly contribute to the sustainable development goals, enhancing women’s health outcomes and preventing cervical cancer.

This study highlights strengths and limitations in identifying factors crucial for the large-scale adoption and implementation of routine cervical cancer screening in South Asia. Employing the CFIR model, the research is pioneering in its application to this context. A comprehensive review of primary literature shows a robust body of work from India, with 37 studies addressing various factors related to cervical cancer screening. However, there is a significant gap in research from Nepal, Pakistan, Bangladesh, Sri Lanka, and other South Asian countries, which lack primary studies on this topic. This underscores the need for additional primary research to explore further the adoption and implementation of cervical cancer screening programs in these regions. This study provides a foundation for future investigations in these critical areas.

## Supplementary Information

Below is the link to the electronic supplementary material.

Supplementary file1 (DOCX 106 KB)

Supplementary file2 (DOCX 20 KB)

Supplementary file3 (DOCX 20 KB)

Supplementary file4 (DOCX 96 KB)

## Data Availability

The data available for this review study are based on publicly accessible sources.

## Abbreviations

AIDS: Acquired immune deficiency syndrome
CFIR: Consolidated Framework for Implementation Research
COVID-19: Coronavirus disease 2019
DNA: Deoxyribonucleic acid
HIV: Human immunodeficiency virus
HPV: Human Papillomavirus
LMIC: Low and middle-income countries
NGOs: Non-governmental organizations
PRISMA: Preferred Reporting Items for Scoping Reviews
SDGs: Sustainable development goals
UHC: Universal Health Coverage
VIA: Visual Inspection with Acetic Acid
VILI: Visual inspection with Lugol’s Iodine
WHO: World Health Organization
