# Supplementary material for: A scoping review of evidence on routine cervical cancer screening in South Asia: investigating factors affecting adoption and implementation
Source: Cancer Causes Control. 2024 Oct 7;36(1):67–79. doi: 10.1007/s10552-024-01923-y (PMC11761498; doi:10.1007/s10552-024-01923-y)
Supplement: Supplementary file 2 — Supplementary file2 (DOCX 20 KB) [file 10552_2024_1923_MOESM2_ESM.docx]

**Annexure 2**

**Search Strategies.** Date of search: 04/01/2023

| **Database name** | **Search strategies/ concepts** | **Hits** |
| --- | --- | --- |
| PubMed | ("cervical cancer screening"[Title/Abstract] OR "cervical screening"[Title/Abstract] OR "routine cervical screening"[Title/Abstract] OR "Uterine Cervical Neoplasms"[MeSH Terms]) AND ("introduc*"[Title/Abstract] OR "implement*"[Title/Abstract] OR "execution"[Title/Abstract] OR "delivery"[Title/Abstract] OR "up take"[Title/Abstract] OR "scaling up"[Title/Abstract] OR "strategy"[Title/Abstract] OR "guidelines"[Title/Abstract] OR "Uptake"[Title/Abstract]) AND ("South Asia"[Title/Abstract] OR "Afghanistan"[Title/Abstract] OR "Bangladesh"[Title/Abstract] OR "Bhutan"[Title/Abstract] OR "India"[Title/Abstract] OR "Maldives"[Title/Abstract] OR "Pakistan"[Title/Abstract] OR "Sri Lanka"[Title/Abstract]) | 250 |
| Web of science | **#1: (((((((TI=(cervical screening)) OR TI=(cervical cancer screening)) OR TI=(routine cervical cancer screening)) OR TI=(routine cervical screening)) OR AB=(cervical screening)) OR AB=(cervical cancer screening)) OR AB=(routine cervical cancer screening)) OR AB=(routine cervical screening)** | [21,238](https://www.webofscience.com/wos/woscc/summary/df1aaa53-cdfe-4af1-a0e9-96e52ea5186e-68b25c30/relevance/1) |
|  | **#2: (((((((((((((((((TI=(implement*)) OR AB=(implement*)) OR AB=(introduct*)) OR TI=(introduct*)) OR TI=(execut*)) OR AB=(execut*)) OR AB=(scale up)) OR TI=(scale up)) OR TI=(scaling up)) OR AB=(scaling up)) OR AB=(delivery)) OR TI=(delivery)) OR TI=(uptake)) OR AB=(uptake)) OR TI=(strateg*)) OR AB=(strateg*)) OR AB=(guideline)) OR TI=(guideline)** | [6,971,795](https://www.webofscience.com/wos/woscc/summary/917e762d-27e8-47d1-b215-9bbff0375779-68b25f77/relevance/1) |
|  | **#3: CU=("INDIA" OR "BANGLADESH" OR "SRI LANKA" OR "PAKISTAN" OR "NEPAL" OR "AFGHANISTAN" OR "MALDIVES" OR "BHUTAN" OR "South Asia")** | [2,155,206](https://www.webofscience.com/wos/woscc/summary/42db9390-8474-4a74-b58b-3fca3c56b9cc-68b26183/relevance/1) |
|  | **#1 AND #2 AND #3** | 264 |
| Scopus | TITLE-ABS ( cervical AND screening ) OR TITLE-ABS ( cervical AND cancer AND screening ) OR TITLE-ABS ( routine AND cervical AND screening ) OR TITLE-ABS ( routine AND cervical AND cancer AND screening ) | 25824 |
|  | TITLE-ABS ( implement* ) OR TITLE-ABS ( delivery ) OR TITLE-ABS ( introduction ) OR TITLE-ABS ( uptake ) OR TITLE-ABS ( scale AND up ) OR TITLE-ABS ( scaling AND up ) OR TITLE-ABS ( executi* ) OR TITLE-ABS-KEY ( guideline ) OR TITLE-ABS ( strategy ) | [10,330,659](https://www.scopus.com/search/history/results.uri?origin=searchhistory&shid=2) |
|  | TITLE-ABS ( south AND asia ) OR TITLE-ABS ( india ) OR TITLE-ABS ( bangladesh ) OR TITLE-ABS ( bhutan ) OR TITLE-ABS ( pakistan ) OR TITLE-ABS ( afghanistan ) OR TITLE-ABS ( maldives ) OR TITLE-ABS ( nepal ) OR TITLE-ABS ( sri AND lanka ) | [679,326](https://www.scopus.com/search/history/results.uri?origin=searchhistory&shid=3) |
|  | ( TITLE-ABS ( cervical  AND screening )  OR  TITLE-ABS ( cervical  AND cancer  AND screening )  OR  TITLE-ABS ( routine  AND cervical  AND screening )  OR  TITLE-ABS ( routine  AND cervical  AND cancer  AND screening ) )  AND  ( TITLE-ABS ( implement* )  OR  TITLE-ABS ( delivery )  OR  TITLE-ABS ( introduction )  OR  TITLE-ABS ( uptake )  OR  TITLE-ABS ( scale  AND  up )  OR  TITLE-ABS ( scaling  AND  up )  OR  TITLE-ABS ( executi* )  OR  TITLE-ABS-KEY ( guideline )  OR  TITLE-ABS ( strategy ) )  AND  ( TITLE-ABS ( south  AND  asia )  OR  TITLE-ABS ( india )  OR  TITLE-ABS ( bangladesh )  OR  TITLE-ABS ( bhutan )  OR  TITLE-ABS ( pakistan )  OR  TITLE-ABS ( afghanistan )  OR  TITLE-ABS ( maldives )  OR  TITLE-ABS ( nepal )  OR  TITLE-ABS ( sri  AND  lanka ) ) | 286 |
| CINAHL | TI ( cervical cancer screening OR cervical screening OR cervical screening programme OR routine cervical scnreening ) OR AB ( cervical cancer screening OR cervical screening OR cervical screening programme OR routine cervical scnreening ) | 5108 |
|  | TI ( implement* OR delivery OR scale up OR uptake ) OR AB ( implement* OR delivery OR scale up OR uptake ) | 408,834 |
|  | TI ( "South Asia" OR Afganistan OR Bangladesh OR Bhutan OR India OR Maldives OR Nepal OR Pakistan OR "Sri Lanka" ) OR AB ( "South Asia" OR Afganistan OR Bangladesh OR Bhutan OR India OR Maldives OR Nepal OR Pakistan OR "Sri Lanka" ) | 49,437 |
|  | (TI "South Asia" OR Afganistan OR Bangladesh OR Bhutan OR India OR Maldives OR Nepal OR Pakistan OR "Sri Lanka" OR AB "South Asia" OR Afganistan OR Bangladesh OR Bhutan OR India OR Maldives OR Nepal OR Pakistan OR "Sri Lanka") AND (S1 AND S2 AND S3) | 37 |
