# Supplementary material for: A scoping review of evidence on routine cervical cancer screening in South Asia: investigating factors affecting adoption and implementation
Source: Cancer Causes Control. 2024 Oct 7;36(1):67–79. doi: 10.1007/s10552-024-01923-y (PMC11761498; doi:10.1007/s10552-024-01923-y)
Supplement: Supplementary file 3 — Supplementary file3 (DOCX 20 KB) [file 10552_2024_1923_MOESM3_ESM.docx]

**Annexure 3**

Data charting form

| **Data Charting form** | |
| --- | --- |
|  | **Information to be filled** |
| Title of the study |  |
| Authors |  |
| Publication year |  |
| Doi |  |
| Journal |  |
| Study Design |  |
| Study Objectives |  |
| Country Name | South Asia: Afghanistan/Bangladesh/  Bhutan/India/Maldives/Nepal/Pakistan/  Sri-Lanka |
| Findings |  |
| Availability of cervical cancer screening services | Yes/ No |
|  | If Yes: Routine/opportunistic |
| Implementation status | Pilot/National/Sub-national |
| Cervical cancer Implementation year (If implemented) |  |
| Positive factors on implementation (If any) |  |
| Negative factors on implementation (If any) |  |
| Positive factors on acceptance/adoption (If any) |  |
| Negative factors on acceptance/adoption (If any) |  |
| Other Key findings (If any) |  |
| Study strengths (If any) |  |
| Study Limitations (If any) |  |
| Conclusion |  |
| Decision (Include/Exclude/Maybe) |  |
| Reason (In the case of exclusion) |  |
