# Supplementary material for: A scoping review of evidence on routine cervical cancer screening in South Asia: investigating factors affecting adoption and implementation
Source: Cancer Causes Control. 2024 Oct 7;36(1):67–79. doi: 10.1007/s10552-024-01923-y (PMC11761498; doi:10.1007/s10552-024-01923-y)
Supplement: Supplementary file 4 — Supplementary file4 (DOCX 96 KB) [file 10552_2024_1923_MOESM4_ESM.docx]

**Annexure 4**

| **Table 3: Characteristics of studies and area of conduct** | | | | | |
| --- | --- | --- | --- | --- | --- |
| **Author** | **Study title** | **Study objective** | **Study design** | **Study origin** | **Findings** |
| (Islam et al., 2015) | Lack of Understanding of Cervical Cancer and Screening Is the Leading Barrier to Screening Uptake in Women at Midlife in Bangladesh: Population-Based Cross-Sectional Survey. | To determine women’s awareness of  cervical cancer and uptake of cervical cancer screening, and to identify socioeconomic, cultural, and religious barriers to screening uptake. | Cross-sectional | Bangladesh | The study found that 81.3% heard about cervical cancer and 48.6% heard about cervical cancer screening. 86% of the women who heard of cervical cancer screening did not do screening as they did not have any symptoms. Significant association was found across having heard of cervical cancer and having been screened with 40–49-year-old, employed out of home, no education and rural dwelling. |
| (Ferdous et al., 2014) | Attitude and practice of cervical cancer screening among the women of Bangladesh. | To determine  the attitude and practice of cervical cancer screening among 401  female doctors of BSMMU. | Cross-sectional | Bangladesh | According to a study, only 40.9% of female doctors had adequate knowledge about cervical cancer screening, and a significant majority of 80.8% had never undergone a screening test. The likelihood of undergoing the test decreased with younger age, lower knowledge scores, and having only a graduate degree. Despite having good knowledge about the importance of the screening test, the study participants showed very low rates of actually getting screened. |
| (Basu et al., 2010) | Evaluation of the National Cervical Cancer Screening Programme of Bangladesh and the formulation of quality assurance guidelines. | To provide a framework for identifying the strengths and  weaknesses of the ongoing programme and to develop a  mechanism to report and resolve problems at the earliest opportunity, To define the measurable indicators that will help assess the performance of the programme in achieving the  stated targets and goals, To assist continuous improvement in quality for all  aspects of cervical screening service delivery. | Evaluation of health programme | Bangladesh | The consultants assessed a screening program and found that it relied mostly on opportunistic screening, but with some elements of organized screening and effective central coordination. However, after four years, the program had low coverage of the targeted population. Patients who underwent colposcopy generally followed through, but almost half of those with high-grade precancers did not receive treatment. The use of cryotherapy was infrequent, and a policy of examining and treating precancers in one visit was rarely implemented. Additionally, the program lacked any internal monitoring or quality control strategies. |
| (Hombaiah et al., 2022) | Effects of mobile Health (mHealth) application on cervical cancer prevention knowledge and screening among women social support groups with low-socioeconomic status in Mysuru city, Southern India. | To assess the effectiveness of the mHealth-based intervention on cervical cancer preparedness among women social support groups. | Interventional study | India | Prior to the intervention, only 13 (12.7%) of the participants had knowledge of cervical cancer. However, following the intervention, there was a notable improvement in their understanding of warning signs, risk factors, and HPV vaccination related to cervical cancer. Additionally, there was a modest increase of approximately 5% in the number of individuals receiving Pap smear tests. |
| (Choudhury & Borah, 2022). | Can financial incentives encourage women to participate in a cervical cancer screening programme? Evidence from a randomized controlled trial analysis. | To evaluate the effectiveness of financial incentives as an intervention to  encourage women to accept and participate in a cervical cancer screening programme. | Randomized controlled trial | India | Individuals who received financial assistance to attend a cancer screening program were significantly more likely to participate, with a likelihood 20.5 times greater compared to those who did not receive financial aid. |
| (Nene et al., 2007). | Determinants of women's participation in cervical cancer screening trial, Maharashtra, India. | To determine the factors associated with participation in cervical cancer screening and follow-up treatment in the context of a randomized controlled trial. | Randomized controlled trial | India | Compared to women who did not undergo screening, those who did were generally younger (between 30-39 years old), more educated, and had a history of using contraception. A greater proportion of screened women were married, while a smaller proportion had never been pregnant. Among the 932 women who were diagnosed with high-grade lesions or invasive cancer, 795 (85.3%) received treatment. Women who had higher levels of education, fewer pregnancies, and were married were more likely to comply with treatment. Analysis of results by the specific screening test received did not reveal any differences in screening rates or compliance with treatment. |
| (Chauhan et al., 2020) | Cost effectiveness of strategies for cervical cancer prevention in India. | To assess the cost effectiveness of various screening strategies for cervical cancer and human papilloma virus (HPV) vaccination in India. | Cost-utility analysis | India | The study examined different cervical cancer screening strategies and found that their implementation resulted in a significant decrease in the occurrence of cervical cancer cases caused by HPV 16/18 and related deaths, compared to no screening. Among the various strategies tested, screening with VIA (visual inspection with acetic acid) every 5 years and every 10 years was found to be cost-effective at one-time per capita GDP, with VIA every 5 years providing greater health benefits. The recommended strategy for India is screening with VIA every 5 years, at an additional cost of US$829 (INR 54,881) per QALY gained. HPV vaccination was also found to be effective, with a 60% reduction in cancer cases and mortality caused by HPV 16/18, compared to no vaccination. When combined with screening (with VIA every 10 years and every 5 years), vaccinated adolescent girls experienced a 69%-76% reduction in cancer cases and a 71%-81% reduction in cancer deaths. Both HPV vaccination alone and vaccination combined with screening (using VIA every 5 years and every 10 years) were deemed cost-effective, with ICERs ranging from US$86 (INR 5,693) to US$476 (INR 31,511) per QALY gained, compared to no vaccination and no screening. |
| (Singh et al., 2014) | Knowledge, attitude and practice of cervical cancer screening in women visiting a tertiary care hospital of Delhi. | To assess the knowledge, attitude and practices of women regarding the basic screening test for detection of cancer cervix. | Cross-sectional | India | Study found that 32.7% of women had adequate knowledge about Pap tests, while only 18.2% had an adequate attitude towards them, and just 7.3% had adequate practices related to them. The main barrier to adequate practice was found to be the lack of request for the test by physicians. The study also found that knowledge, attitudes, and practices increased significantly with age and education levels. |
| (Zutshi et al., 2017) | Cervical Cancer Screening and Prevention: An Analysis of Beliefs and Predictors of Knowledge, Attitude and Practice in Northern India. | To access awareness of cervical cancer, its screening, HPV infection and its vac- cination in women attending tertiary care hospital in north India. | Cross-sectional | India | The majority of women (73%) were aware that cervical cancer is preventable, and 58.6% knew about screening tests for it. However, only 3% of women had undergone screening. Most women had become aware of cervical cancer and screening through visits to doctors. Only a small percentage (2%) knew about the link between HPV infection and cervical cancer, but almost all women (99%) were willing to get vaccinated if it was available for free. |
| (Kulkarni et al., 2022) | Compliance for Community-Based Cervical Cancer Screening Program among Women of Age 30-65 Years Residing in Low Socioeconomic Settings of Mumbai, India. | To study factors determining compliance of women for cervical cancer screening in an urban low socioeconomic setting. | Cross-sectional | India | The following factors were found to be associated with higher participation in screening for cervical cancer: women in the age group of 30-39 years old (80.69%), those who were literate with education up to Senior College level (78.97% and 80.86%), women who were Hindu (77.20%), those who spoke Marathi (77.07%), and those who had a family history of cancer (81.93%). On the other hand, women who were Muslim (73.95%), those who spoke a language other than Marathi and Hindi (73%) , illiterate women (70.71%), and women with a graduate degree (70.78%) had lower participation rates. |
| (Sharma et al., 2018) | Feasibility of implementing cervical cancer screening program using smartphone imaging as a training aid for nurses in rural India. | To assess the relaibility and feasibility of cervical cancer screeing by healthworkers/ nurses using VIA in rural setting, To assess the role of smart phone imaging for continous trainig of nurses | Cross-sectional | India | The nurse identified 25 cases as VIA-positive (indicating the presence of cervical abnormalities), but only 19 of them sought further medical attention. The kappa statistic, a measure of agreement between the nurse and the expert physician, was 0.45 (with a 95% confidence interval of 0.26-0.63), indicating a moderate level of agreement. The expert's evaluation was affected by image retrieval and quality issues. The implementation of VIA screening faced challenges such as low awareness among the population and difficulties in linking up patients with appropriate follow-up care. |
| (Poli et al., 2020) | Feasibility, Acceptability, and Efficacy of a Community Health Worker-Driven Approach to Screen Hard-to-Reach Periurban Women Using Self-Sampled HPV Detection Test in India. | To evaluate the feasibility, acceptability, and efficacy of implementing an HPV self-sampling–based approach to screen a socioeconomically disadvantaged, unscreened population. | Cross-sectional | India | After receiving counseling from community health workers (CHWs), women were willing to provide self-collected samples. The screening test showed a positivity rate of 6.4%, and CHWs successfully guided 65% of HPV-positive women for further examination through colposcopy. The rate of detecting cervical intraepithelial neoplasia (CIN) 2+ was 9.7 per 1,000 women screened. The HPV test showed a positive predictive value of 15.3% to detect CIN 2+ lesions. Triage with visual inspection using acetic acid significantly improved the positive predictive value to 49.5% to detect CIN 2+ lesions, but missed detecting a significant number of CIN 2+ lesions. The sensitivity of colposcopy was suboptimal. Among 51 women with lesions, 80% underwent ablative treatment, and most of them received same-day treatment. |
| (Khanna et al., 2019) | Knowledge, attitude, and practice about cervical cancer and its screening among community healthcare workers of Varanasi district, Uttar Pradesh, India. | To assess the socio demographic profile and KAP for cervical cancer and its screening of community health workers. | Cross-sectional | India | The study found that although workers had good knowledge and perception about screening, less than 10% had undergone screening. The level of knowledge was significantly associated with the practice of screening. |
| (Narayana et al., 2017) | Knowledge, attitude, and practice toward cervical cancer among women attending Obstetrics and Gynecology Department: A cross-sectional, hospital-based survey in South India. | To assess knowledge, attitude,  and practice (KAP) toward cervical cancer, screening, and prevention | Cross-sectional | India | The study found that among the respondents, 74.6% had heard of cervical cancer, with media and friends being the primary sources of information. A majority of the women were aware of the symptoms, risk factors, screening methods, and preventive measures for cervical cancer. More than half of the women had a positive attitude toward screening, but 86.6% did not have a practice of undergoing cervical cancer screening. Sociodemographic factors were strongly linked to levels of knowledge, attitude, and practice regarding cervical cancer. |
| (Joshi et al., 2021) | Mobile Screening Unit (MSU) for the Implementation of the 'Screen and Treat' Programme for Cervical Cancer Prevention In Pune, India. | Evaluation of an opportunistic point of care cervical cancer screening initiative  in Pune, India using a mobile screening unit (MSU). | Cross-sectional | India | A total of 10,925 women were screened for cervical cancer in 290 outreach clinics in the MSU. The overall positivity rate was 6.6%, with a decreasing trend over time. Out of 717 women who were eligible for treatment, 304 (42.4%) received thermal ablation, and 11 of them (3.6%) reported minor side effects, while 5 (1.6%) experienced lower abdominal pain, which resolved after treatment. Of the 413 women advised to undergo colposcopy, only 20.33% did so. Of the 84 women who underwent the procedure, 64 had normal results, while 7 had CIN1, 2 had CIN2, 9 had CIN3, and 2 were diagnosed with invasive cancer. |
| (Swapnajaswanth et al., 2014) | Perception and Practices on Screening and Vaccination for Carcinoma Cervix among Female Healthcare Professional in Tertiary Care Hospitals in Bangalore, India. | To elicit information on the knowledge, attitude and practice (KAP) regarding screening (Pap test) and vaccination for carcinoma cervix among female doctors and nurses in a tertiary care hospital in Bangalore and to assess barriers to acceptance of the Pap test. | cross-sectional | India | The study found that a greater percentage of doctors (45 or 78.9%) had very good knowledge about the risk factors for cervical cancer and Pap tests, compared to only 13 nurses (13.3%) with the same level of knowledge. This difference was statistically significant (p=0.001). The majority of the study participants (138 or 89.6%) had a positive attitude towards Pap tests and vaccination. However, a significant proportion of them (114 or 73.6%) had never undergone a Pap test. The most common reason for not practicing Pap testing was the absence of disease symptoms, reported by 35 participants (31%). |
| (Srivastava et al., 2022) | Prevalence and Predictors of Cervical Cancer Screening among Reproductive Age Group Women: Evidence from Cross-Sectional Study in Rohtak and Delhi. | To estimate the prevalence and determine the factors for cervical cancer screening among women in the reproductive age group in Delhi and Rohtak, India. | Cross-sectional | India | Approximately 35.2% of the respondents had knowledge about cervical cancer screening, with a higher percentage in Delhi than in Rohtak. Only 3.9% had undergone screening for cervical cancer, with a higher percentage in Haryana than in Delhi. Women who had knowledge about cervical cancer were more likely to undergo screening. Women over 30 years of age had significantly higher odds of undergoing cervical cancer screening than women aged 30 years or less. Women from households with a monthly income of more than 15000 had significantly higher odds of undergoing cervical cancer screening than women from households with an income of 5000 or less. |
| (Mulmi et al., 2022) | Community-Based Cervical Cancer Screening using Visual Inspection with Acetic Acid | To assess the prevalence of precancerous lesions of cervical cancer from community-based screening programs in Nepal | Cross-sectional study | India | During the study period, a total of 7,270 women were screened, and 153 of them (2.1%) were found to have cervical precancerous lesions. The highest prevalence of such lesions was observed in women aged 30 and below, with 46 (3%) testing positive. Among the total women screened, the majority (61%) were married before the age of 20, and 188 (3%) reported a family history of cancer. The most common symptoms reported by the women were low abdominal pain (17%), low back pain (16%), itching in the anogenital region (11%), and vaginal discharge (11%). Cervical cancer was suspected in 25 (0.3%) of the women. |
| (Pimple & Shastri, 2014) | Comparative evaluation of human papilloma virus-DNA test verses colposcopy as secondary cervical cancer screening test to triage screen positive women on primary screening by visual inspection with 5% Acetic acid | Evaluates the performance of secondary testing by human papillomavirus (HPV) test and Colposcopy in a single-visit screening approach to increase program effectiveness in limited health-care resources settings. | Cross-sectional study | India | The study included 3613 women, out of which 352 (9.7%) tested positive for cervical intraepithelial neoplasia grade 2+ on primary screening using VIA. The VIA test showed a sensitivity of 93% (95% confidence interval (CI): 0.76-0.99) and a specificity of 91% (95% CI: 0.90-0.92) for detecting cervical intraepithelial neoplasia grade 2+ lesions. HPV DNA and colposcopy were used as secondary tests for triaging VIA-positive women, and they showed a sensitivity of 61% (95% CI: 0.41-0.78) and 43% (95% CI: 0.24-0.63), respectively, for detecting CIN2+ lesions. Both HPV DNA and colposcopy had a specificity of 99% (95% CI: 0.99-1.00 and 0.99-0.99, respectively) for detecting CIN2+ lesions. |
| (Sodhani et al., 2006) | Test characteristics of various screening modalities for cervical cancer: a feasibility study to develop an alternative strategy for resource-limited settings | To determine test characteristics – sensitivity, specificity, positive and negative predictive values – of  different screening modalities to detect cervical precancerous and cancerous lesions in order to devise an effective  alternative strategy for cervical cancer screening in resource-poor settings. | Cross-sectional | India | The study showed The sensitivity of VIA was lower (86.7% compared to 91.4%), but its specificity was higher (90.7% versus 86.6%) than cytology when assessing for low-grade squamous intraepithelial lesions (LSIL). However, this difference did not reach statistical significance (P > 0.01). HPV testing increased the sensitivity compared to cytology (97.1% versus 91.4%), but there was a non-significant decrease in specificity (84.2% versus 86.6%). The results of VIAM were relatively similar to those of VIA. |
| (Agarwal et al., 2022) | Attitude and Perceived Barriers Among Highly Educated Women Towards Cervical Cancer Screening by Pap Smear: An Online Survey. | Attitude and perceived barriers among  highly educated women who can understand the significance of timely cervical screening to etermine the  association between the socio-demographic characteristics and their attitude towards cervical cancer  screening by Pap smear | Cross-sectional | India | The findings indicated that 150 women took part in the study, and their average age was 36.9+9.7 years. The majority of women (85.33%) showed no apparent symptoms. Overall, most participants (82.67%) held a positive view regarding cervical cancer screening, but only a small percentage (5.33%) had actually undergone screening in the past. One significant barrier to proper screening practice was the perception that a Pap test was not necessary. Furthermore, the study revealed a significant association between women's attitudes towards screening and factors such as age, marital status, and education level. |
| (Chandrika et al., 2020) | Awareness on cancer cervix, willingness, and barriers for screening of cancer cervix among women: A community-based cross-sectional study from urban Pondicherry | To assess the awareness about cervical cancer, willingness, and barriers for undergoing screening of cervical cancer among women in urban Pondicherry. | Cross-sectional | India | Approximately one-third of women had knowledge about cervical cancer. Women who were younger, had higher levels of education, had a family history of cancer, and were currently employed demonstrated higher levels of awareness. However, awareness regarding the risk factors, signs, and symptoms of cervical cancer was low. While 60% of women who were aware of cervical cancer recognized the possibility of early detection, less than 15% were familiar with the various methods available for early detection. Among the participants, 32% expressed willingness to undergo cervical cancer screening, and occupation, family history of cancer, and knowledge about risk factors were identified as independent factors associated with this willingness. The major reasons for unwillingness to undergo screening were fear and the belief of not having signs and symptoms. |
| (Hariprasad et al., 2020) | Evaluation of a Chip-Based, Point-of-Care, Portable, Real-Time Micro PCR Analyzer for the Detection of High-Risk Human Papillomavirus in Uterine Cervix in India. | To determine if the diagnostic performance of Truenat, a point-of-care, portable, battery-operated HPV DNA testing device that detects 4 high-risk HPV genotypes (16, 18, 31, and 45) is comparable to the reference standard test, Hybrid Capture 2 (HC2) for cervical HPV DNA testing. | Cross-sectional | India | The research findings revealed that out of 615 cervical samples, the HR-HPV DNA test yielded positive results in 78 women (12.7%) using HC2 and in 49 women (8%) using Truenat. When taking into account the limited range of genotypes included, the Truenat HPV-HR test demonstrated a sensitivity of 97.7% & specificity of 98.9%. |
| (Patil et al., 2019) | Perspectives of primary healthcare providers on implementing cancer screening services in tribal block of Maharashtra, India. | To understand the  willingness, knowledge, barriers, and facilitating factors in implementing cancer screening services in tribal block of Maharastra, among primary healthcare providers | Interventional (Pre and Post) | India | The findings indicated that there was a high level of knowledge about the causes of common cancers; however, awareness regarding specific risk factors was low. There were noticeable gaps in knowledge regarding HPV vaccination. Following the continuing medical education (CME), there was an overall improvement in understanding the available screening methods and knowledge about the HPV vaccine and its recommended dosages (pretest 65% to posttest 95%). Providers lacked experience in performing routine cervical cancer screenings. Although the majority of providers (97%) recognized the importance and feasibility of cancer screening at the primary healthcare (PHC) level, training (52%) and resources (53%) were identified as necessary requirements. |
| (Bhatt et al., 2018) | Mobile technology and cancer screening: Lessons from rural India. | Analysis of uptake in response to screening and follow-up invitations, complemented by qualitative data from 8 key informant interviews and 2 focus groups | Qualitative | India | A total of 8,686 individuals underwent screening using the mHealth intervention, with the majority (98%) being screened for oral cancer. The positivity rate for cervical screening was 28%, and out of those who tested positive, 37% attended for follow-up. For oral cancer screening, the positivity rate was 5%, and 31% of those who tested positive attended for follow-up. Community health workers (CHWs) found the mHealth prototype highly acceptable and believed it enhanced the reliability of the screening process. Several obstacles to screening and follow-up in individuals who tested positive were identified. The use of the mHealth prototype positively impacted the social status of the CHWs involved in delivering the interventions. |
| (Mahalakshmi & Suresh, 2020) | Barriers to cancer screening uptake in women: A qualitative study from Tamil Nadu, India | Experiences and perceptions of cancer screening were explored with particular focus on barriers to screening uptake and possible solutions. | Qualitative | India | The average age of the individuals involved in the study was 38 years. Among the participants, 38.9% underwent breast cancer screening, while 16.7% underwent cervical cancer screening. Psychosocial factors, such as the fear of the screening procedure and the fear of being diagnosed with cancer, emerged as significant barriers to screening uptake. Other contributing factors included a lack of awareness, cultural beliefs, financial constraints, and issues related to the healthcare system. Participants suggested that changes in government policies to implement mandatory screening programs, the provision of incentives, and increased awareness campaigns could facilitate higher screening rates among women. |
| (Mahalakshmi & Suresh, 2020) | Cervical cancer screening status and implementation challenges: Report from selected states of India. | To explore the challenges encountered by program managers and health workers at district level when implementing the NPCDCS program, looking specifically at the perceived capacity of the health system to offer accessible screening services. | Qualitative | India | The findings indicated that participants expressed a perception of inadequate capacity across all six domains for the nationwide implementation of the CCS program. To overcome the barriers related to the health system, potential solutions include implementing context-specific strategies, improving coordination between the program and district health facilities, ensuring timely remuneration, enhancing data maintenance practices, and establishing a robust monitoring system. |
| (Jyoshma Preema Dsouza et al., 2020) | Exploring the Barriers to Cervical Cancer Screening through the Lens of Implementers and Beneficiaries of the National Screening Program: A Multi-Contextual Study. | To identify the specific barriers that prevent the uptake of cervical cancer screening. | Qualitative | India | The general health concern among the participants was low, and routine check-ups were considered unimportant. Poor knowledge about cervical cancer, benefits of screening service availability, as well as a general sense of well-being, embarrassment or anxiety related to the screening procedure, fear of being judged for lack of modesty, and stigma were common barriers to screening uptake. In addition to a general unawareness of cervical cancer geographical inaccessibility of screening as a barrier to participate in cervical cancer screening, in certain regions. |
| (Shastri et al., 2014) | Effect of VIA Screening by Primary Health Workers: Randomized Controlled Study in Mumbai, India | To investigate the feasibility and efficacy of VIA performed by trained primary health workers (PHWs) in reducing cervical cancer mortality among women aged 35 to 64 years living in Mumbai, India. | Randomized controlled trial | India | In the group that underwent screening, we observed a screening participation rate of 89% and a diagnosis confirmation compliance rate of 79.4%. Among those diagnosed with invasive cancer, the treatment compliance rate was 86.3% in the screening group and 72.3% in the control group. Notably, the screening group exhibited a statistically significant 31% reduction in cervical cancer mortality (relative risk = 0.69; 95% confidence interval = 0.54 to 0.88; p = 0.003). |
| (Shaki et al., 2018) | A study on cervical cancer screening in asymptomatic women using Papanicolaou smear in a tertiary care hospital in an urban area of Mumbai, India. | To detect early cervical neoplasia in asymptomatic women of educated society living in urban  locality. | Cross-sectional | India | Out of a total of 1100 cases, the majority consisted of benign findings, including 581 (52.8%) cases classified as negative for intraepithelial neoplasia (NILM), 203 (18.4%) cases categorized as inflammatory, and 74 (6%) cases identified as high-grade squamous intraepithelial lesion (HSIL). The overall sensitivity and specificity for detecting low-grade squamous intraepithelial lesion (LSIL) were 75.8% and 94.6% respectively, while the sensitivity and specificity for detecting HSIL were 68.9% and 98.6% respectively. |
| (Dhanasekaran et al., 2019) | Cervical Cancer Screening Services at Tertiary Healthcare Facility: An Alternative Approach. | To study the  association between socio-demographics and abnormal  cytology results on Pap test population screened  and to assess the usefulness of the model satellite cervical  cancer screening clinic run by paramedical staff. | Secondary data analysis | India | Out of the 5,328 women who underwent screening, 2% (96 out of 5,328) had abnormal cytology results, including cases of malignancy. Notably, there was a statistically significant positive association between abnormal cytology and Muslim women (p<0.05). The study's conclusion emphasizes the urgent need for increased awareness regarding cervical cancer screening in resource-limited countries. Additionally, the recommendation is made for government hospitals in such countries to establish dedicated preventive oncology units specifically for conducting cancer screenings. |
| (Dhillon et al., 2020) | Is India's public health care system prepared for cervical cancer screening?: Evaluating facility readiness from the fourth round of the District Level Household and Facility Survey (DLHS-4) | To assess facility readiness for cervical cancer screening at all four levels of the health care system prior to the initiation of universal screening of common NCDs in 2016 utilizing data from the nationally representative using DLHS 4 data (2012-13). | Secondary data analysis | India | The study revealed that overall readiness scores for cervical cancer screening were generally low. Among the subcategories, primary health centers (PHCs) had low scores in 'potential staffing' due to limited manpower for diagnosis and treatment (cryotherapy) of potential cases. At secondary health centers (SHCs), the lowest scores were observed in 'infrastructure' and 'infection prevention'. However, scores were higher for tiers involved in diagnostic work-up and treatment/referral. Notably, 'potential staffing' received the highest scores across all tiers, except for PHCs. On the other hand, 'infection prevention' and 'medicines and laboratory' obtained the lowest scores. In terms of regional variations, Goa and Maharashtra consistently ranked among the top five states with higher readiness scores. |
| (Sen et al., 2022) | Socio-economic and regional variation in breast and cervical cancer screening among Indian women of reproductive age: a study from National Family Health Survey, 2019-21 | To examine the socio-economic and regional variations of breast and cervical screening among Indian women in the reproductive age. | Secondary data analysis | India | The study findings indicate that breast and cervical cancer screening rates were 877 and 1965 per 100,000 women, respectively, among women aged 30-49. However, the screening rates were lower among women who were poor, young, had lower levels of education, and lived in rural areas. The concentration index, which measures inequality, was 0.2 for ever screening of breast cancer and 0.15 for cervical cancer among women aged 30-49. The concentration curve for both breast and cervical cancer screening indicated a pro-rich pattern, indicating higher screening rates among wealthier individuals. Factors such as higher educational attainment, being aged 40-49, and residing in the western or southern regions were associated with significantly higher odds of undergoing screening for breast or cervical cancer. The patterns of screening for breast and cervical cancer among women aged 15-49 were found to be similar to those observed among women aged 30-49. |
| (George T, 2021) | Factors influencing utilization of cervical cancer screening services among women: A cross sectional survey | To assess various factors affecting utilization of cervical cancer screening services. | Cross-sectional | India | The findings of the study revealed that there was a remarkably low utilization of cervical cancer screening services among the population under investigation, with only 5% of individuals having ever undergone a Pap smear screening in their lifetime. A significant association was observed between a lower level of knowledge regarding cervical cancer screening and nonparticipation in screening services. Several factors were identified as important barriers to the utilization of cervical cancer screening services, including lack of awareness (25.06%), inadequate availability of healthcare facilities (22.67%), absence of symptoms (11.69%), perception of low risk (11.93%), and social stigma (9.55%). These findings highlight the critical need for increased awareness and improved accessibility to healthcare facilities in order to enhance the utilization of cervical cancer screening services in the studied population. |
| (Seeta Devi, 2020) | Influencing reasons for participation and nonparticipation of women in cervical screening | To find influencing reasons for participation and nonparticipation for cervical screening among three experimental groups. 2. To find the views of women regarding verbal, written and video based health education on prevention and early detection of cervical cancer. | Quasi experimental | India | The study findings revealed that a significant portion of participants across the three experimental groups, approximately 38.32% in the verbal group, 41.91% in the written group, and 40.71% in the video group, expressed their inability to participate in the cervical screening program due to feeling highly embarrassed about the procedure. Other participants mentioned reasons such as considering themselves healthy and therefore not needing the test, having only one sexual partner which they believed made the test unnecessary, assuming the test to be expensive, not experiencing any symptoms of cancer and thus not seeing the need for the Pap test, and lacking knowledge about the recommended frequency of the test. |
| (Subba et al., 2022) | Knowledge and attitude towards, and the utilisation of cervical and breast cancer screening services by female healthcare professionals at a tertiary care hospital of Eastern India: A cross-sectional study | knowledge and utilisation of screening methods for cervical and breast cancer and to elaborate barriers for non-utilisation amongst female HCPs, both doctors and nurses | Cross-sectional | India | The study findings indicated that a low percentage of respondents, specifically 26.5% for clinical breast examination and 7% for mammography, reported practicing these screening methods. Furthermore, a minimal number of healthcare professionals, comprising only seven doctors and one nurse, had undergone Pap smear testing. On a positive note, more than 90% of the respondents demonstrated knowledge of the risk factors, signs and symptoms, as well as the availability of screening tools for detecting cervical and breast carcinoma. |
| (Ramtel et al., 2022) | Factors associated with utilization of visual inspection with acetic acid in Nepal | This research aimed to  identify and describe the factors that are associated with the utilization of VIA among women in Nepal so  that it helps to increase the uptake of screening services. | cross- sectional | India | The study findings revealed that the utilization of VIA screening in the province was influenced by several factors, including awareness, economic status, socio-psychological beliefs, service availability and accessibility, and perception of service providers' performance. Socio-demographic variables and knowledge factors were also found to be associated with VIA screening. The approach of service providers during the VIA screening process played a significant role in the acceptance of this service. Additionally, there was a notable disparity in women's knowledge of cervical cancer and screening. |
| (Darj et al., 2019) | Barriers and facilitators to cervical cancer screening in Nepal: A qualitative study | to investigate and better understand Nepali women’s perceptions on barriers to participation in cervical cancer screening and what might facilitate their participation. | Qulitative | Nepal | Women have lack of awareness of the screening and misconceptions about it. Attending screening centres was viewed as being difficult due to sociocultural barriers, service providers' actions, geographic difficulties, and a lack of resources. Encouragement from family and women's groups, as well as participation in awareness campaigns, may persuade women to go to screening clinics. |
| (Greibe Andersen et al., 2020) | Barriers and facilitators to cervical cancer screening uptake among women in Nepal: a qualitative study | The objective of this qualitative study was to investigate barriers and facilitators to screening participation among Nepalese women aged 30–60 years. | Qualitative | Nepal | This generated five key themes: 1) lack of spouse's backing for screening, 2) universal stigma and prejudice, 3) lack of understanding about screening alternatives, 4) getting examined, and 5) health care professionals. We advise decision-makers and interested parties to use these findings to increase awareness, information access, and higher-quality screening programmes in Nepal. |
| (Shrestha et al., 2021) | Feasibility of HPV self-sampling pathway in Kathmandu Valley, Nepal using a human-centred design approach. | The present study aims to explore the feasibility and acceptability of a self-sampling-based approach for cervical cancer screening in urban and peri-urban Nepal and develop pathways for self-sampling using a co-design methodology | Qualitative | Nepal | Most individuals did not have any knowledge about HPV and its connection to cervical cancer. There was a widespread lack of faith in internet buying, despite the fact that 70% of respondents (n = 21/31) had done so in the past. The majority of the women (n = 17/30; 56.7%) showed a readiness to self-sample and gave suggestions on how to make the instructions more clear. The suggested user experience was deemed practical in an urban setting. In Nepal, there is a definite unmet need for knowledge regarding HPV and alternate cervical screening methods. |
| (Thapa et al., 2018) | Knowledge, attitude, practice and barriers of cervical cancer screening among women living in mid-western rural, Nepal | To find out the knowledge, attitude, practice, and barriers of cervical cancer screening in mid-western rural, Nepal. | Cross-sectional | Nepal | The study included 360 participants, with a mean age of 30.13 10.4 years. Over 87 percent of people lacked sufficient understanding, although roughly 72% supported cervical cancer screening. Eighty-four percent of the women had never undergone a cervical cancer screening test. Despite having greater literacy rates than Dalits and Janajatis, Brahmins and Chhetris were less likely to go to a cervical cancer screening (p 0.001); additionally, those with a strong family history of cancer were more likely to go (p 0.001). Similar to this, married women were more likely to practise cervical cancer screening if they had sufficient knowledge and/or a positive attitude, however this was statistically insignificant. |
| (Majid et al., 2022) | Awareness, screening, practices and attitudes of cervical cancer among doctors and nursing staff working at a tertiary care centre. | To assess knowledge, attitude and practice of medical and paramedical staff about cervical cancer as well as its screening and prevention. | Cross-sectional | Pakistan | Among the 347 participants, 144 (41.5% of them nurses) and 203 (58.5% of them doctors) were present. In general, people were 26.22 6.38 years old. 108 (or 30%) of the total respondents were married, whereas 239 (or 68%), were not. Overall, 239 people (68.8%) were aware that the Pap smear is a screening test; 85 people (24.5%) knew the real guidelines for repeating the test; 152 people (43.8%) knew how exactly to use the visual inspection with acetic acid; 61 people (17.6%) had had a Pap smear performed; and 156 people (45%) believed they were at risk of developing cervix cancer. Multiple sexual partners 254 (73.2%), age at first sex 160 (46%), smoking 131 (37.8%), offensive discharge 221 (63.7%), and post-coital bleeding 231 (66.6%) were also common risk factors. |
| (Shivanthan et al., 2014) | Low uptake of Pap smear testing among medical clinic attendees in a tertiary care hospital in Sri Lanka. | To determine the knowledge, attitudes and practices related to Pap smear testing among women attending a medical clinic in a tertiary care hospital in Sri Lanka, specifically looking at factors which influence uptake of the test. | Cross-sectional | Sri Lanka | 188 of the 280 eligible women that were interviewed (or 67.1%) in total. The ages of the participants ranged from 29 to 65. 111 women (59.0%) were aware of Pap smear testing, however 41 of them (36.9%) were unaware that the test may identify pre-cancerous lesions. Only 34 patients (18.1%) had ever had a Pap smear performed. Prior knowledge of the test (p=0.001), higher parity (p=0.022), and knowing someone with cervical cancer (p=0.001) were all univariate associated with Pap smear uptake. The same independent relationships of test uptake were verified by multivariate regression analysis (p=0.001, 0.012, and 0.013, respectively). |
| (K. C. M. Perera et al., 2021) | The feasibility of new HPV/DNA test as a primary cervical cancer screening method among 35-years- old ever-married women in Kalutara district; a cross-sectional study | Determine the feasibility of a new HPV/DNA screening test among 35- year age cohort of ever- married women in a district of Sri Lanka. | Cross-sectional | Sri Lanka. | The overall response rate was 91.1%. Customers' opinions on the HPV/DNA screening test process were overwhelmingly favourable (99.9%, n = 821) and 99.6% (n = 819) of customers stated that it would be beneficial for the test to be included in the National Cervical Cancer Screening programme. HPV was present in 6.2% of people (95% CI: 6.18-6.22%). The HPV/DNA screening had an 89.5% coverage rate (n = 822). 0.12% of the stated results were unreliable (n = 1). When compared to the number of women who attempted to get a colposcopy within a month of being referred, the percentage of women with positive HPV/DNA test results who performed a pap test within 3 months of the initial screening was 100% (n = 51). |
| (Witharana et al., 2015) | Awareness of female malignancies among women and their partners in Southern Sri Lanka and implications for screening: a cross sectional study | The objectives of this study were a) to assess the awareness of symptoms, risk factors and available screening facilities for three common female malignancies, namely; breast, cervical and uterine malignancies among women and their male partners, b) to assess the percentage utilization of screening services and self-examination strategies for early detection by women, c) to correlate utilization of health services by women with awareness, level of education and acceptance of screening by male partner, and d) to correlate utilization of health services by women with level of education, family income and employment status of women. | Cross-sectional | Sri Lanka | Interviews were conducted with a total of 282 couples (n-282, 564 persons). All types of cancers were poorly understood. On a measure measuring awareness, more than 50% of participants from both sexes received less than half the marks. Better awareness was significantly associated with better family income, education, and stable work in both sexes. In certain cases, better participation was correlated with encouragement from the male spouse. |
| (K. C. Perera et al., 2022) | Acceptability, simplicity, and relevance of the new human papillomavirus/ DNA test among 35-year-old ever-married women in a district of Sri Lanka: focus group discussions | The objective of the study was to explore the acceptability, relevance, and simplicity of new HPV/DNA screening implementation as a primary cervical cancer screening method among 35 -year old ever-married women in a district of Sri Lanka. | Qualitative | Sri Lanka | The majority of participants (n = 17, 70.9%), Buddhists (n = 18, n = 75%), and non-workers (n = 18, n = 75%) were Sinhalese. Most of the participants expressed their great appreciation for the field staff's performance and the community's awareness of HPV/DNA screening. Most people were aware of the HPV/DNA test's great sensitivity, which results in a high percentage of early diagnosis of cervical cancer precursors. The majority of participants described the HPV/DNA test as neutral and convenient. In HPV/DNA screened positive follow-up, it was frequently reported that further clinic visits were required for the pap test and colposcopy, although there was a pronounced acceptability (n = 23, 95.8%) for the HPV/DNA test. |
| (Khatuja et al., 2022) | Knowledge and practice of cervical cancer screening in general population and medical personnel: A gap to be bridged. | To evaluate the knowledge, attitude, and practice among women attending the outpatient department (OPD) and medical personnel | Cross-sectional | India | Women in the wider community knew and understood 32.5% about cervical cancer and its screening, compared to 100% of medical professionals, whereas only 25% of general population women actually had their cervixes screened. Lack of information was the main barrier to practise for the general public, while for medical professionals, it was due to hesitation, shame, a lack of time, and a lack of strong motivation. |
| (Saiyed et al., 2020) | Community level barriers for cervical cancer screening in marginalized population | To understand theknowledge/awareness and practices of the community, tounderstand the predictors of cervical cancer screening,and to assess the acceptance of the HPV test in the community. | Cross-sectional | India | It was shown that community women lacked awareness of cervical cancer and screening practises. The value of preventative healthcare is not well understood, and evidence-based practises are almost nonexistent. Sociodemographic traits are crucial indicators of whether someone would participate in the screening programme. |
| (Ali et al., 2010) | Knowledge and awareness about cervical cancer and its prevention amongst interns and nursing staff in Tertiary Care Hospitals in Karachi, Pakistan. | To assess the knowledge and awareness about cervical cancer and its prevention amongst health professionals in tertiary care hospitals in Karachi, Pakistan. | Cross-sectional | Pakistan | 1.8% of people who were interviewed did not realise that cervical cancer is a disease. Only 23.3% of those polled understood that cervical cancer is the leading cause of gynaecological cancers and that it also has the second-highest fatality rate. Infection is the most frequent cause of cervical cancer, and 78% of respondents were aware of this fact. Of these, 62% believed that a virus was to blame, and 61% understood that the virus in question was the Human Papilloma Virus (HPV). Only a minority (41%) knew that HPV may be detected by PCR, but the majority were aware that it is sexually transmitted. Only 26% of the participants in the study were aware of any risk factors. 37 percent of respondents acknowledged the Pap smear as a screening test. |
| (J P Dsouza et al., 2022) | A comparison of behavioural models  explaining cervical cancer screening uptake. | To explore determinants of screening uptake | Cross-sectional | India | The primary explanation for CCS (cervical cancer screening) intention was effectively provided by the Theory of Planned Behavior (TPB) and subsequently supported by the Health Belief Model (HBM). Among the factors considered in these models, the factors that had the greatest impact on screening intention were having a positive attitude towards the screening procedure and perceiving the benefits it offers. They were followed by emotions such as fear, anxiety, or embarrassment related to the disease or the screening procedure, as well as barriers specific to the context. |
| (Patra et al., 2017) | Awareness of cervical cancer and willingness to participate in screening program: Public health policy implications | To find out awareness about cervical cancer among women and their willingness to utilize screening services in an urban resettlement colony of Delhi, India | Cross-sectional | India | The study comprised a sample of 373 women, with an average age of 39.14 years. Approximately two-thirds of the participants had no formal education. Only half of the participants had knowledge of cervical cancer, and merely a quarter of them expressed a willingness to undergo a screening test. The inclination to participate in screening was more pronounced among those with a higher level of education, individuals who had used family planning methods in the past, and those who possessed knowledge about at least one risk factor, signs or symptoms, or the potential for early detection of cervical cancer. |
| (Ghosh et al., 2021) | Knowledge, Attitude and Practices Towards Cervical Cancer and its Screening Among Women from Tribal Population: a Community-Based Study from Southern India | To assess knowledge, attitude and practices (KAP) towards cervical cancer screening among tribal women of coastal Karnataka, southern India | Cross-sectional | India | The average age of the participants was 39.8 ± 10.1 years. While 82.9% of the participants reported being familiar with cervical cancer, only 51% were aware of its preventability, and a mere 2.3% knew about the possibility of early detection. More than 75% of the participants lacked sufficient knowledge about cervical cancer. However, the majority of them (99.9%) held a positive attitude towards cervical cancer screening. None of the participants had undergone cervical cancer screening prior to the study. The participants' knowledge scores were significantly associated with variables such as age group, marital status, education level, socioeconomic status, and tribal community (p < 0.05). |
| (Vidhubala et al., 2020) | Loss to follow-up after initial screening for cervical cancer  A qualitative exploration of barriers in Southern India. | To explore the barriers to community-based screening and suggest possible solutions for mitigating the loss to follow-up among screen-positive women from the perspectives of both, the community women (CWs) and service providers (SPs) | Qualitative | India | Lack of awareness and limited comprehension of the screening process, fear related to both the procedures and the disease itself, absence of financial and family support, and adherence to sociocultural beliefs were identified as obstacles to follow-up. To address this, the study recommended to maintain consistent follow-up by utilizing phone calls and home visits, while simultaneously increasing awareness about the screening process and its advantages. Building a strong connection with the community and leveraging local community-based organizations to track individuals are proposed as potential strategies to reduce the rate of loss to follow-up. |
| (Yadav et al., 2022) | A cross-sectional study on assessment of perceived threat to cervical cancer using health belief model among women in a slum area of Kolkata. | A cross-sectional study on assessment of perceived threat to cervical cancer using health belief model among women in a slum area of Kolkata. | Cross-sectional | India | The study found that 22.9% of the participants exhibited satisfactory levels of perceived threat towards cervical cancer. A statistically significant correlation was observed between unsatisfactory levels of perceived threat and certain factors, including being in the reproductive age group (adjusted odds ratio [AOR] = 3.01; P = 0.036), having an education level up to primary (AOR = 2.89; P = 0.026), and possessing insufficient knowledge (AOR = 2.94; P < 0.001) among the respondents. The multivariable regression model demonstrated a good fit. |
| (Ahmed et al., 2008) | Development of a visual inspection programme for cervical cancer prevention in Bangladesh. | To train Government of Bangladesh service providers to perform VIA for cervical screening for early detection of cervical cancer and pre-cancerous conditions, develop referral linkages to ensure follow-up and management of women with cervical intraepithelial neoplasia (CIN), to motivate other health personnel and communities to support the setting up of a cervical cancer screening programme. | Development of health program | Bangladesh | The assessment of the pilot program demonstrated that trained healthcare professionals, including doctors, nurses, and paramedical workers, in Bangladesh could successfully conduct VIA despite limited resources and a lack of awareness among women, their partners, and families about the disease and its consequences. In order to enhance the program's effectiveness, the study recommended that it is necessary to transition from opportunistic screening to a population-based, systematic screening approach targeting women aged 30 and above. This requires additional training for more healthcare providers and improving the infrastructure of clinics. Moreover, it was found that there is a need to strengthen the connections between screening, diagnosis, and treatment, while also significantly reducing the false-positive rate of VIA tests. |

| **Table 4:** factors influencing the implementation and adoption of routine cervical cancer screening in South Asia. | | | | | |
| --- | --- | --- | --- | --- | --- |
| **Sl No** | **Country** | **No of articles** | **Positive factors on adoption and implementation** | **Negative factors on adoption and implementation** | **Other factors** |
| 1 | Bangladesh | 4 | -availability of screening and opportunistic screening methods  -Keen interest in utilizing screening services in women, education level  -Active participation of community healthcare workers dissemination events increased the screening participation | -Lack of symptoms and screening knowledge among women with lower education levels and residing in rural areas  -lack of healthcare infrastructure to avail the services  -Scarcity of trained health professionals to screen | -Cultural or religious beliefs |
| 2 | India | 37 | -Availability of resources, trained staff, cost-effective screening test  -Willingness of healthcare staff to undergo training  -Good knowledge of cervical cancer, screening, and vaccination among women  -Govt programs, policies, and incentives | - lack of advice by physicians  -Absence of disease symptoms  -Limited access to healthcare  -Pain associated with the test, fear, modesty concerns, and financial constraints | -social, cultural factors and taboo  - High cost of HPV DNA and pap test  - Longer waiting hours for screening in the hospital |
| 3 | Nepal | 5 | - Establishment of the National Cervical Cancer Screening and Prevention Guideline  -Low-cost and self-sampling kit | -Perception of having no symptoms to get screened  -Long waiting hours for screening  - Lack of knowledge, myths, poverty, fear, limited accessibility, privacy concerns, and lack of support from family members | -Family history of cancer participated more in screening |
| 4 | Pakistan | 2 | - Healthcare students are aware of the availability of screening tests | - Limited knowledge and negative attitudes among healthcare staff  - Socioeconomic disparities in certain regions, lower education level | -Insufficient understanding of cervical cancer's prevalence |
| 5 | Sri Lanka | 4 | - Role played by the media, awareness about screening tests  -Awareness campaigns and effective field staff performance | - Unmarried women not getting screened due to stigma  - The fear of discomfort during screening | -Inadequate screening coverage among healthcare coverage |
